# Frailty Parameters, Morbidity and Mortality in Older Adults with Cancer: A Structural Equation Modelling Approach Based on the Fried Phenotype

**DOI:** 10.3390/jcm9061826

**Published:** 2020-06-11

**Authors:** Frederic Pamoukdjian, Marie Laurent, Claudia Martinez-Tapia, Yves Rolland, Elena Paillaud, Florence Canoui-Poitrine

**Affiliations:** 1 APHP, Avicenne Hospital, Geriatric Department, Coordination Unit in Geriatric Oncology, F-93000 Bobigny, France; 2 Univ Paris Est Creteil, INSERM, IMRB, F-94010 Creteil, France; marie.laurent@aphp.fr (M.L.); claudia.martinez-tapia@aphp.fr (C.M.-T.); elena.paillaud@aphp.fr (E.P.); florence.canoui-poitrine@aphp.fr (F.C.-P.); 3 APHP, Henri-Mondor Hospital, Internal Medicine and Geriatric Department, Paris-Sud-Val-de-Marne Geriatric Oncology Clinic, F-94000 Créteil, France; 4 Gérontopôle de Toulouse, Institut du Vieillissement, Centre Hospitalo-Universitaire de Toulouse, 37 Allée Jules Guesde, 31000 Toulouse, France; rolland.y@chu-toulouse.fr; 5 UPS/INSERM UMR 1027, University of Toulouse III, Toulouse, France Faculté de Médecine, 37 Allées Jules Guesde, 31000 Toulouse, France; 6 APHP, Paris Cancer Institute CARPEM, Geriatric Oncology Unit, European Georges Pompidou Hospital, F-75015 Paris, France; 7 APHP, Henri-Mondor Hospital, Public Health Department, F-94000 Créteil, France

**Keywords:** sarcopenia, cachexia, cancer, structural equation modelling, mortality, morbidity

## Abstract

Background: to distinguish direct and indirect pathways to frailty phenotype, and quantify associations between two frailty components (i.e., sarcopenia and cachexia) regarding mortality and morbidity in older adults with cancer. Methods: all consecutive older outpatients with cancer were included in a prospective two-centre cohort study between 2013 and 2017 and had geriatric assessment. We used the frailty phenotype. Sarcopenia and cachexia were built as latent variables by including observed variables related to physical performances and related to nutrition and inflammation respectively. Structural equation modelling was used to distinguish between direct and indirect effects of the frailty parameters on the risk of death (Model 1) and the risk of morbidity (defined by unplanned hospitalization and/or disability and/or a fall; Model 2). The root mean square error of approximation (RMSEA) and the comparative fit index (CFI) were used to assess the model fit. Results: 603 older outpatients were included (mean age: 81.2 ± 6.1; women: 54%; frailty phenotype: 58%). The 6-month mortality and morbidity rates were 18% and 64%, respectively. The fit was good for both models (RMSEA and CFI = 0.029 [0.017–0.039] and 0.99 for Model 1, and 0.028 [0.017–0.039] and 0.99 for Model 2, respectively). Sarcopenia and cachexia were both directly and significantly associated with 6-month mortality (β^sarcopenia^ = 0.18, *p* = 0.01; β^cachexia^ = 0.52, *p* < 0.0001) and morbidity (β^sarcopenia^ = 0.37, *p* < 0.0001; β^cachexia^ = 0.19, *p* < 0.02). Conclusions: sarcopenia and cachexia had a direct pathway with 6-month mortality and morbidity in older cancer patients.

## 1. Introduction

In older adults, frailty is defined as a state of high vulnerability to stressors. It is associated with a number of poor health outcomes, including early mortality, disability, falls, and unplanned hospitalization [1]. Of the various indices of frailty developed over the last 20 years [2], one of the most frequently used for research purposes is Fried’s frailty phenotype (based on five physical frailty criteria: shrinking, exhaustion, low physical activity, slowness, and weakness) [3], for which a pathophysiological model has been suggested [2].

Given the complexity of the pathophysiological links between the frailty phenotype’s components, we reasoned that structural equation modelling (SEM) could usefully (i) distinguish direct and indirect pathways to frailty, and (ii) quantify associations between frailty components [4]. Thus, the frailty phenotype corresponds to a vicious circle centred on sarcopenia (due to comorbidities and pathological aging) which leads to exhaustion and a decrease in mobility (slowness), strength (weakness), physical activity, and overall energy expenditure—thus strengthening a chronic state of undernutrition. In turn, chronic undernutrition worsens the sarcopenia [3]. Sarcopenia is the age-associated loss of skeletal muscle mass, muscle strength and/or physical performances [5]. Cachexia is an ongoing loss of skeletal muscle mass that cannot be fully reversed by conventional nutritional support and that leads to progressive functional impairment [6].

Since cancer is a strong stressor and a prevalent disease in older adults, it is an appropriate pathological situation in which to characterize pathways leading to the frailty phenotype and the related poor health outcomes [7]. To the best of our knowledge, the pathophysiological model of the frailty phenotype was not validated in older adults with cancer.

Hence, we hypothesized that the Fried frailty phenotype is applicable to older patients with cancer, and that SEM could distinguish between the direct and indirect effects of frailty parameters, and especially sarcopenia and cachexia, on morbidity and mortality in this population. We sought to assess the plausibility of this pathophysiological model for explaining early death, disability, falls and unplanned hospitalization in older adults with cancer by using an SEM approach.

## 2. Methods

### 2.1. Study Design and Population

From 15 November 2013 to 30 September 2017, outpatient members of the French Physical Frailty in Elderly Cancer (PF-EC) cohort were screened for inclusion in the present study. The PF-EC prospective, observational, two-centre cohort study has been described in detail elsewhere [8]. Briefly, all consecutive older in- and outpatients referred for a geriatric assessment (GA) at two university hospitals in the greater Paris area were prospectively included in the PF-EC cohort once a diagnosis of cancer had been established, and before the cancer treatment decision had been made. For the purposes of the present study, we excluded inpatients because walking test data were not usually available for this subgroup (e.g., because infusions therapy prevented the performance of walking tests).

The inclusion date was considered to be the date of the patient’s first consultation in the participating geriatric oncology department.

All participants provided their informed consent before inclusion in the study. The study was approved by the local independent ethics committee (Avicenne Hospital, Bobigny, France; reference: CLEA-2015-019).

### 2.2. Cancer-Related and Demographic Data

Demographic data (age and sex), cancer-related data (tumour site and extension: local, locally advanced, i.e., a non-resectable tumour with no distant metastases, or metastatic) were recorded at the first geriatric oncology consultation, as part of the geriatric assessment (GA). The type of treatment received by each patient was categorized as either supportive care alone or another type of care, and was recorded during the 6-month follow-up consultation.

### 2.3. Geriatric Assessment (GA)

The GA was performed during the patient’s first consultation in the geriatric oncology department. Comorbidities were assessed using the Cumulative Illness Rating Scale for Geriatrics (CIRS(G)). Total comorbidity burden was defined as a total CIRS(G) score above the median value of 14 [9]. Dependency was defined as a six-item activities of daily living (ADL) score below or equal to 5 out of 6, and/or by a four-item simplified instrumental ADL score (IADL: using the telephone, transport, medications, and money management) of less than 4 out of 4 [10,11]. Malnutrition was defined as a body mass index (BMI) below 21 kg/m^2^ [12]. Impaired mobility was defined as a short physical performance battery (SPPB) score below 9 out of 12 and/or a one-leg stance balance test time below 5 s [13,14]. Depressed mood was defined as a Mini-Geriatric Depression Scale (Mini-GDS) score of 1 or more out of 4 [15]. Cognitive impairment was defined as a Mini-Mental State Examination (MMSE) score below 24 out of 30 [16].

### 2.4. Definition of Frailty

Data on the frailty phenotype were collected during the patient’s GA [3,17]. Frailty was defined as at least three of five criteria developed by Fried et al.

Shrinking was defined by an unintentional loss weight of at least 5% in the previous year.

Self-report of exhaustion (i.e., poor endurance and energy) was identified by two questions: “I felt that everything I did was an effort” and “I could not get going”. Patients were asked “How often in the last week did you feel this way?” 0 = rarely or none of the time; 1 = some or a little of the time; 2 = a moderate amount of the time; or 3 = most of the time. Exhaustion was defined as an answer of “2” or “3” to either of these questions.

Low physical activity was defined by a single question. Individuals who denied doing daily leisure activities (such as walking or gardening) and/or sports activity at least once a week were categorized as physically inactive [18].

Slowness was defined as a slow gait speed, according to the sex- and height-adjusted cut-off values established by Fried et al. Gait speed (in m/s) was measured with a stopwatch for participants walking over a short distance (4 m, along a corridor) at their usual pace.

Weakness was defined as an impairment in maximum hand grip strength (in kilograms), according to the sex- and BMI-adjusted cut-off values established by Fried et al. Hand grip strength was measured twice for each hand using a hand-held dynamometer (EH101, Camry Electronic Ltd., Zhaoqing, China).

## 3. Covariates

Negative protein balance was assessed as the serum albumin level (measured in an immunoturbidimetric assay) over the 3 weeks immediately after the GA. A value below 35 g/L was considered to be abnormal [12]. Inflammation was assessed in terms of the serum C-reactive protein (CRP) level (measured in an immunoturbidimetric assay) over the 3 weeks immediately after the GA. A value above 10 mg/L was considered to be abnormal [19].

### 3.1. Adverse Health Outcomes

Each patient had a follow-up consultation every three months during the first year after inclusion.

The main outcome was mortality in the 6 months after the GA. Vital status was determined by telephoning the patients or their family or by extracting data from medical records.

The secondary outcome was morbidity in the 6 months after the GA. This was a composite outcome defined as at least one of the following frailty-related events: the first unplanned hospitalization and/or disability (defined by the loss of at least 1 point on the ADL and/or IADL scales), and/or a fall.

### 3.2. Prespecified Structural Equation Modelling (SEM) of the Frailty Phenotype

**Figure 1:**
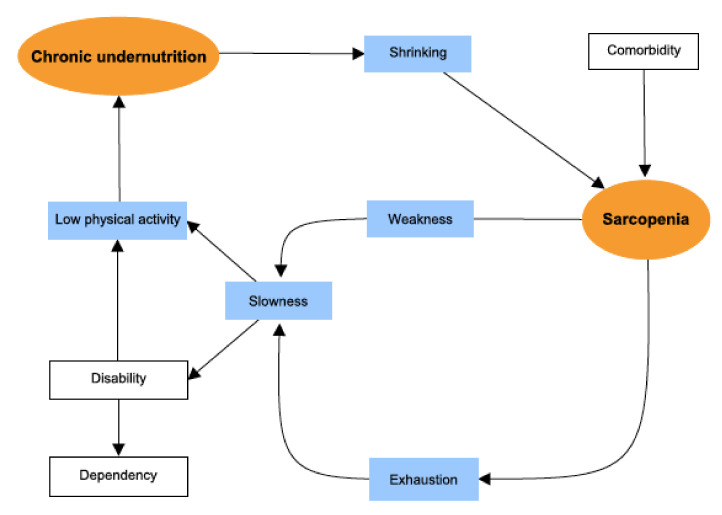
Pre-specified structural equation modelling (SEM) of the pathophysiology cycle for frailty. By convention, circles (orange) represent latent variables and rectangles represent observed variables. Blue rectangle = Frailty criteria. The direction of the arrow indicates the hypothetical pathway, as proposed by Fried et al.

Structural equation modelling is based on the analysis of both observed and non-observed (i.e., latent) variables or constructs. The analysis uses a combination of correlation measures and regressions to assess the direct and indirect pathways within a pre-specified pathophysiological model [20]. Figure 1 shows the pre-specified structural equation modelling for the pathophysiology of the frailty phenotype adapted from Fried et al.’s theoretical model.

The central factor in the pathophysiological model is sarcopenia—the age-associated loss of skeletal muscle mass (quantitative impairment), muscle strength and/or physical performance (qualitative impairment) [5]. Due to the absence of a direct measurement of muscle mass in our study, sarcopenia was defined as a latent variable by including observed variables related to physical performances. Thus, we used low physical activity, slowness, weakness, one-leg stance balance test, and the SPPB score to define sarcopenia. For application to our population of patients with cancer, chronic undernutrition was used as a proxy for cachexia. The latter syndrome is defined as an ongoing loss of skeletal muscle mass that cannot be fully reversed by conventional nutritional support and that leads to progressive functional impairment. The pathophysiology of cachexia is characterized by a negative protein and energy balance (i.e., undernutrition) driven by a variable combination of reduced food intake and excessive catabolism (i.e., inflammation) [6]. Again, due to the absence of a direct measurement of muscle mass in our study, cachexia was defined as a latent variable by including observed variables related to nutrition and inflammation (shrinking, BMI, serum albumin level, and serum CRP level). Exhaustion was not used to build the latent variables because we suspected that it significantly influenced the relationship between cachexia and sarcopenia. This is in line with the pathophysiological model provided by Fried et al., in which sarcopenia induced exhaustion. The other observed variables were considered for adjustment of the structural equation model of the frailty phenotype.

With the exception of the cancer site and cancer extension, each observed variable was binary (yes/no, with cut-off values defined above).

### 3.3. Statistical Analyses

Descriptive analysis: categorical variables were summarized as the number (percentage), and continuous variables were summarized as the mean ± standard deviation (SD) or the median [interquartile range (IQR)].

Correlations: we first built a correlation matrix for each observed variable by using Cramer’s V test (as is appropriate for a correlation between two qualitative variables).

Confirmatory factor analysis (CFA) [20]: the two latent variables were included in a CFA. The “diagonally weighted least squares” estimator was used to obtain estimates with standard errors and standardized coefficients.

Structural equation modelling [20]: the relationships between latent variables and endogenous variables were expressed as standardized coefficients (equivalent to the correlation coefficient). Linear or logistic regression was used to assess direct and indirect interactions between the two latent variables, as appropriate. Using a multivariate logistic regression analysis, two structural equation models (Models 1 and 2) were finally fitted to estimate the total direct effects of several predictors (i.e., sarcopenia + cancer cachexia + cancer site + cancer extension) for the two outcomes assessed (i.e., Model 1 for mortality, and Model 2 for morbidity). The result of the regression was expressed as the odds ratio (OR) by exponentiation of the standardized coefficients (equivalent to the β coefficients).

Model fit [20]: we used two indices to characterize the quality of the CFA and the structural equation models. Firstly, the root mean square error of approximation (RMSEA) and its confidence interval (CI) describe the complexity of the model assessed. An RMSEA <0.05 was considered to be adequate. Secondly, the comparative fit index (CFI) compares the tested models with an independent model. A CFI >0.95 was considered to be adequate.

All tests were two-sided, and the threshold for statistical significance was set to *p* < 0.05. The data were analysed using R statistical software (version 3.4.3, R Foundation for Statistical Computing, Vienna, Austria, http://www.R-project.org). Multivariate imputation by chained equations was used to handle missing data for shrinking (*n* = 9), exhaustion (*n* = 1), low physical activity (*n* = 1), slowness (*n* = 3), weakness (*n* = 8), BMI (*n* = 3), SPPB (*n* = 2), one-leg stance balance (*n* = 1), serum CRP (*n* = 112), and serum albumin (*n* = 100) via the MICE package in R. Structural equation modelling was performed by using the Lavaan R package.

## 4. Results

### 4.1. Patients

**Figure 2:**
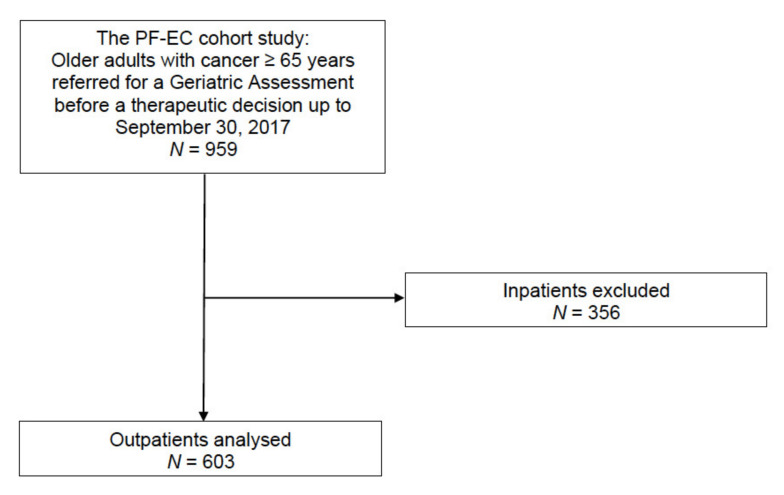
Flow chart.

Of the 959 consecutive older (aged 65 and over) patients with cancer in the PF-EC cohort having been referred for a GA up until 30 September 2017, 356 were excluded because they were inpatients. Hence, 603 outpatients were included in the present study (Figure 2).

### 4.2. Baseline Characteristics of Patients and the Frailty Phenotype

**Figure 3:**
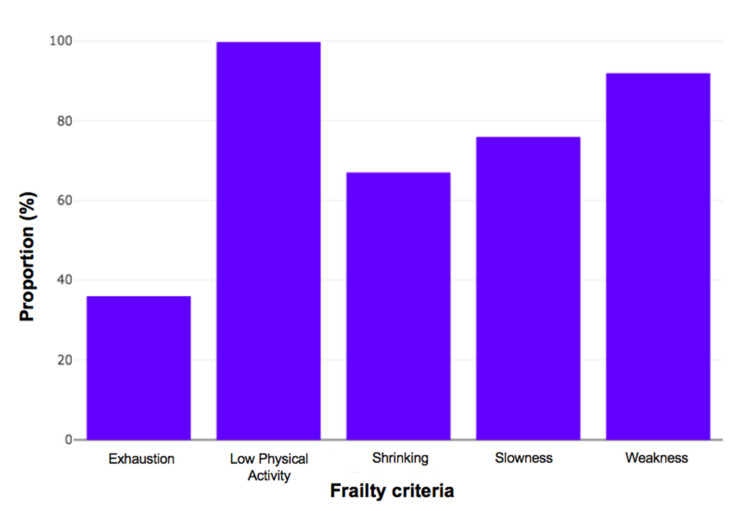
Proportion of frailty criteria in 352 frail older patients with cancer.

Table 1 shows the baseline characteristics of the included patients. The mean ± SD age of the study population was 81.2 ± 6.1 years. Most of the patients were women (54%), with solid tumours (94%) and locally advanced (38%) or metastatic cancer (45%). Colorectal and breast cancers were the two most common types. The majority of the patients were frail. Figure 3 shows the proportion of patients with frailty criteria in the subgroup of frail patients: exhaustion (36%), shrinking (66.9%), slowness (75.9%), weakness (91.9%), and low physical activity (99.7%). The proportion of patients with impairments in the GA varied from 14% to 64%, depending on the domain and the thresholds used.

### 4.3. Correlations between Frailty Criteria and the Other Observed Variables

Table 2 shows the correlation matrix for the observed qualitative variables. Shrinking was positively correlated with exhaustion, BMI, serum albumin, and serum CRP. Low physical activity was positively correlated with slowness, weakness, the one-leg stance balance and the SPPB score. Exhaustion was positively correlated with slowness and the SPPB score. The correlations were consistent with the pre-specified latent variables.

### 4.4. Goodness of Fit

In a CFA, we first tested the adequacy of the two latent variables (sarcopenia and cachexia). The model’s fit was good, with an RMSEA (CI) of 0.032 (0.012–0.049) and a CFI of 0.99. There was significant covariance between the latent variables (standardized coefficient: 0.32; *p* < 0.0001). When we added exhaustion to each latent variable, the model’s fit worsened: the RMSEA (CI) and a CFI were respectively 0.054 (0.041–0.067) and 0.98 for sarcopenia; and 0.056 (0.043–0.069) and 0.98 for cachexia. Thus, we chose to keep exhaustion as an exogenous mediating variable for the two latent variables.

### 4.5. Pathways of the Frailty Phenotype Leading to Mortality

**Figure 4:**
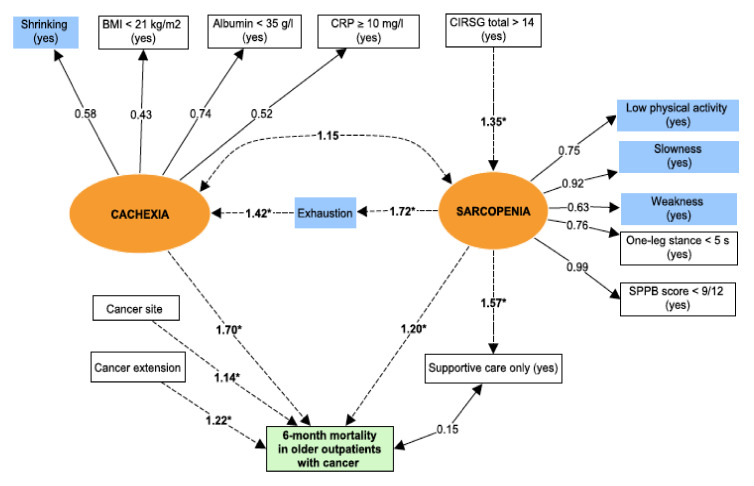
The pathways of the frailty phenotype leading to mortality. Circles (orange) = latent variables; Rectangles = observed variables; Correlations: dotted lines; Regressions: solid lines; Covariance: * *p* value (for regressions) < 0.05; Blue rectangle = Frailty criteria; Green rectangle = Outcome. Latent variables represent non-observed variables but which summarize a set of directly observed variables. Latent variables are constructed by using strong correlations between observed variables. Regressions are then used to assess association between latent variables and outcomes.

The mortality rate (95% confidence interval (CI)) 6 months after the initial GA was 17.9% (14.9–21.2) (*n* = 108 out of 603). The structural equation modelling of the relationship between frailty and mortality had a good fit: the RMSEA was 0.029 (0.017–0.039), and the CFI was 0.99. The path diagram for the structural equation model 1 is summarized in Figure 4. The total comorbidity burden was significantly associated with sarcopenia, while age and sex were not (data not included in Figure 4). While there was no direct association between the latent variables sarcopenia and cachexia, both were significantly linked to exhaustion. Sarcopenia and cachexia were both associated (independently of the cancer site and extension) with 6-month mortality in older outpatients with cancer. Cachexia had the strongest association with mortality. The provision of supportive care was not associated with mortality but was significantly linked to the sarcopenia variable. The path coefficients for regressions in the SEM are detailed in Table 3.

### 4.6. Frailty Phenotype Pathways Leading to Morbidity

**Figure 5:**
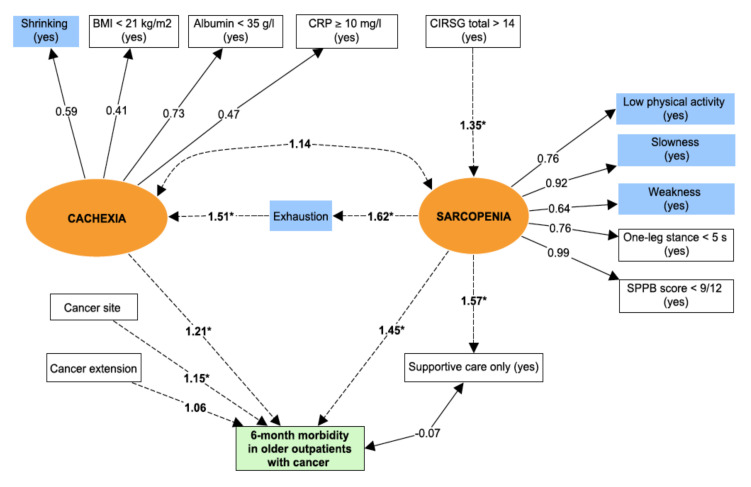
The pathways of the frailty phenotype leading to. Circles (orange) = latent variables; Rectangles = observed variables; Correlations: dotted lines; Regressions: solid lines; Covariance: * *p* value (for regressions) < 0.05; Blue rectangle = Frailty criteria; Green rectangle = Outcome. Latent variables represent non-observed variables but which summarize a set of directly observed variables. Latent variables are constructed by using strong correlations between observed variables. Regressions are then used to assess association between latent variables and outcomes.

The morbidity rate [95% CI] 6 months after the initial GA was 64% (60–68) (*n* = 386 out of 603). Unplanned hospitalization affected 24% (*n* = 146/603) of the patients, disability affected 48% (*n* = 289 out of 603); and falls affected 18.5% (*n* = 112 out of 603). Again, the fit for the structural equation modelling of the relationship between frailty and morbidity was good: the RMSEA was 0.028 (0.017–0.039), and the CFI was 0.99. The path diagram for the structural equation model 2 is summarized in Figure 5. We obtained substantially the same results as for Model 1 (Table 3). Here, sarcopenia had the strongest association with morbidity. Cancer extension was not independently associated with morbidity.

## 5. Discussion

We external validated the pathophysiological model of the Fried’s frailty phenotype in older patients with cancer. We confirmed the plausibility and the consistency of this model with regard to 6-month morbidity (unplanned hospitalization and/or disability and/or fall) and mortality. The latent variables sarcopenia and cachexia were both directly associated with 6-month mortality and morbidity. Cachexia had the strongest association with mortality, and sarcopenia had the strongest association with morbidity. Exhaustion was found to significantly influence the relationship between sarcopenia and cachexia, and cachexia was found to be the end stage of frailty.

Understanding the pathophysiology of frailty is a key issue in preventing related complications in older patients with cancer. Here, we provided a comprehensive model of the frailty phenotype centred on sarcopenia and cachexia in older patients with cancer. Sarcopenia and cachexia are highly prevalent in cancer patients (particularly in older adults), with a prevalence of between 10% and 75% for sarcopenia and between 25% and 80% for cachexia (depending on the cancer site/extension) [21,22]. In line with our results, although both sarcopenia and cachexia involve muscle wasting, the former appears to be more strongly related to impaired physical performances, and the latter appears to be more related to malnutrition and inflammation [5,6]. However, the two syndromes may be at opposite ends of a continuum (starting with sarcopenia and ending with cachexia) mediated by exhaustion. In line with our findings, exhaustion was found to be significantly associated with sarcopenia in the context of cachexia in two recent observational studies [4,23].

As described above, we found that the comorbidity burden was directly associated with sarcopenia in each of our two models; indeed, comorbidities are well-known risk factors for sarcopenia [5]. However, we found that cancer-related data were not directly associated with the pathophysiology of frailty but were independently associated with mortality and morbidity. Hence, the frailty phenotype does not appear to be influenced by comorbidities such as cancer [3].

Our study had several strengths. First, we longitudinally assessed four frailty-related outcomes (i.e., mortality, unplanned hospitalization, disability, and falls) via regular patient monitoring. Second, the study is the first (to the best of our knowledge) to have externally validated the pathophysiology of frailty in older patients with cancer. Third, our analysis was based on a robust methodology (SEM) that enabled us to understand the nature of the pathological pathways leading to the frailty phenotype.

The main limitation of our study was probably its overestimation of the prevalence of frailty. First, the oncologists, surgeons and other physicians had referred patients who they already suspected to be frail. Moreover, the modified version of the frailty phenotype could lead also to a measurement bias. Another limit is the relative high number of missing data for albumin and CRP could lead to selection and classification biases which were minimized by multiple imputation. At last, although muscle mass was not directly measured, our model remains plausible by summarizing a set of directly observed variables related to physical performances.

Our study confirms the value of using the frailty phenotype to define frailty in older patients with cancer in routine clinical practice; the phenotype is based on a solid pathophysiological model, and can potentially be reversed by target geriatric interventions [24]. In particular, our study results show that sarcopenia and cachexia are risk factors for mortality and morbidity in older cancer patients. Moreover, our model suggests a direct pathway from comorbidities to sarcopenia. It is known that rehabilitation approaches had to be multidisciplinary for improving muscle mass (i.e., dietary, physical activity with resistance training) [25,26,27]. Our model suggests that it may also be multi-domain by targeting comorbidities as well [28,29]. A further validation of our study results would be needed with a longer follow-up.

## 6. Conclusions

We used SEM to confirm the plausibility and the consistency of the pathophysiological model of frailty established by Fried et al. Sarcopenia and cachexia were directly associated with 6-month mortality and morbidity in older patients with cancer. Exhaustion was a mediating variable between sarcopenia and cachexia.

## Figures and Tables

**Table 1:** Baseline characteristics of the study population (*n* = 603).

Variables	*n* (%) or Median [IQR]
Age (year)	82 [77–86]
Sex (female)	323 (54)
Frailty phenotype (yes)	352 (58)
Cancer site	
Colorectal	109 (18)
Breast	105 (17)
Lung	92 (15)
Liver	85 (14)
Digestive tract other than colorectal ^a^	79 (13)
Genito-urinary tract	40 (7)
Haematological malignancies	34 (6)
Skin with melanoma	16 (2.5)
Prostatic	16 (2.5)
Others ^b^	27 (5)
Cancer extension	
Local	104 (17)
Locally advanced	228 (38)
Metastatic	271 (45)
Supportive care only (yes)	124 (20.5)
Comorbidities (CIRS(G)):	
Total >14	269 (45)
Dependence	
ADL £ 5/6	204 (34)
IADL £ 3/4	386 (64)
Nutrition	
BMI < 21 kg/m^2^	84 (14)
Serum albumin level < 35 g/L	221 (37)
Serum CRP ≥ 10 mg/L	267 (44)
Mobility	
SPPB < 9/12	314 (52)
One-leg stance balance < 5 s	448 (74)
Mood	
Mini-GDS ≥ 1/4	261 (44)
Missing data	6 (1)
Cognition	
MMSE < 24/30	217 (51)
Missing data	174 (29)

^a^: pancreas (*n* = 28), gastric (*n* = 20), bile-duct (*n* = 15), oesophagus (*n* = 9), gastrointestinal stroma tumour (*n* = 4), anal (*n* = 3); ^b^: unknown primary site (*n* = 10), mesothelioma (*n* = 8), sarcoma (*n* = 5), head and neck (*n* = 3), thymoma (*n* = 1). ADL: activities of daily living; BMI: body mass index; CIRS(G): Cumulative Illness Rating Scale for Geriatrics; CRP: C-reactive protein; ECOG-PS: Eastern Cooperative Oncology Group performance status; GS: gait speed; IADL: instrumental activities of daily living; Mini-GDS: Mini-Geriatric Depression Scale; MMSE: Mini-Mental State Examination; SPPB: short physical performance battery.

**Table 2:** Correlation matrix for observed variables.

	Shrinking (Yes)	Exhaustion (Yes)	Low Physical Activity (Yes)	Slowness (Yes)	Weakness (Yes)	One-Leg Stance < 5 s (Yes)	SPPB < 9/12 (Yes)	BMI < 21 kg/m^2^ (Yes)	Albumin < 35 g/L (Yes)	CRP ≥ 10 mg/L (Yes)	Age ≥ 82 Years (Yes)	CIRS(G) Total > 14 (Yes)	Cancer Site	Cancer Extension	Supportive Care Only (Yes)
Shrinking (yes)	1														
Exhaustion (yes)	0.24 *	1													
Low physical Activity (yes)	0.13 *	0.13 *	1												
Slowness (yes)	0.10 *	0.31 *	0.27 *	1											
Weakness (yes)	0.06	0.18 *	0.23 *	0.34 *	1										
One-leg stance < 5 s (yes)	0.06	0.15 *	0.33 *	0.37 *	0.35 *	1									
SPPB < 9/12 (yes)	0.09 *	0.29 *	0.29 *	0.75 *	0.38 *	0.50 *	1								
BMI < 21 kg/m^2^ (yes)	0.18 *	0.18 *	0.04	0.05	0.03	0.06	0.03	1							
Serum albumin < 35 g/L (yes)	0.22 *	0.20 *	0.10 *	0.21 *	0.14 *	0.13 *	0.20 *	0.17 *	1						
Serum CRP ≥ 10 mg/L (yes)	0.20 *	0.12 *	0.08 *	0.05	0.05	0.06	0.06	0.09 *	0.30 *	1					
Age ≥ 82 years (yes)	0.06	0.05	0.08 *	0.16 *	0.15 *	0.20 *	0.24 *	0.09 *	0.04	0.003	1				
CIRS(G) total > 14 (yes)	0.01	0.05	0.11 *	0.25 *	0.15 *	0.19 *	0.25 *	0.11 *	0.11 *	0.10 *	0.14 *	1			
Cancer site	0.30 *	0.12	0.15	0.21 *	0.14	0.14	0.20 *	0.15	0.20 *	0.25 *	0.19 *	0.12	1		
Cancer extension	0.08	0.05	0.02	0.02	0.02	0.04	0.02	0.02	0.04	0.15 *	0.09	0.02	0.24 *	1	
Supportive care only (yes)	0.03	0.15 *	0.08 *	0.25 *	0.17 *	0.14 *	0.23 *	0.09 *	0.13 *	0.05	0.14 *	0.09 *	0.23 *	0.08	1

* *p* value < 0.05 (Cramer’s V test). SPPB: short physical performance battery; BMI: body mass index; CRP: C-reactive protein; CIRS(G) = Cumulative Illness Rating Scale for Geriatrics.

**Table 3:** Path coefficients estimated using regression (linear or logistic) in a structural equation model of the relationship between frailty components, mortality and morbidity in older outpatients with cancer.

Models	Estimate	Standard Error	Standardized Coefficient (β Coefficient)	*p* Value
**Model 1**				
Sarcopenia~CIRS(G) total > 14	0.64	0.1	0.30	<0.0001
Exhaustion~sarcopenia	0.47	0.06	0.47	<0.0001
Cachexia~exhaustion	0.16	0.05	0.37	0.001
Mortality *				
~ sarcopenia	0.18	0.07	0.18	0.01
~ cachexia	1.30	0.29	0.52	<0.0001
~ cancer site	0.05	0.02	0.13	0.03
~ cancer extension	0.23	0.07	0.20	<0.0001
Supportive care only ~ sarcopenia	0.44	0.06	0.45	<0.0001
**Model 2**				
Sarcopenia ~ CIRS(G) total > 14	0.64	0.1	0.30	<0.0001
Exhaustion ~ sarcopenia	0.47	0.06	0.48	<0.0001
Cachexia ~ exhaustion	0.17	0.05	0.41	<0.0001
Morbidity *				
~ sarcopenia	0.36	0.06	0.37	<0.0001
~ cachexia	0.46	0.20	0.19	0.02
~ cancer site	0.05	0.02	0.14	0.01
~ cancer extension	0.06	0.06	0.06	0.28
Supportive care only ~ sarcopenia	0.44	0.06	0.45	<0.0001

* Multivariate analysis; ~ = regression; CIRS(G) = Cumulative Illness Rating Scale for Geriatrics.
